# Plasma ferritin, C-reactive protein, and adenosine deaminase levels in tuberculous lymphadenitis and pleuritis and their role in monitoring treatment response

**DOI:** 10.1186/s12879-024-10228-z

**Published:** 2024-12-02

**Authors:** Zaib un Nisa, Basit Zeshan, Atiqa Ambreen, Tehmina Mustafa

**Affiliations:** 1 Department of Pathology, Gulab Devi Hospital, Lahore, Pakistan; 2 Department of Microbiology, Faculty of Science & Technology, University of Central Punjab, Lahore, Pakistan; 3 Faculty of Sustainable Agriculture, Universiti Malaysia Sabah, 90000 Sandakan, Sabah Malaysia; 4 Department of Microbiology, Gulab Devi Hospital, Lahore, Pakistan; 5 Center for International Health, Department of Global Public Health and Primary Care, University of Bergen, P.O box 7804, N-5020 Bergen, Norway; 6 Department of Thoracic Medicine, Haukeland University Hospital, Bergen, Norway

**Keywords:** CRP, Ferritin, ADA, Treatment response, EPTB, TB lymphadenitis, TB pleuritis

## Abstract

**Background:**

We aimed to assess the plasma levels of ferritin, C-reactive protein (CRP), and adenosine deaminase (ADA) at baseline and their utility as biomarkers to monitor response to treatment in extrapulmonary tuberculosis (EPTB) patients.

**Methods:**

Prospective measurements of ferritin, CRP, and ADA were done in unstimulated plasma samples of 92 EPTB (49 TB lymphadenitis and 43 TB pleuritis) patients registered for anti-TB treatment. Blood samples were taken at the start, 2, and 6 months of treatment, plasma levels of ferritin and CRP were measured by the enzyme-linked immunosorbent assay and ADA levels by kinetic chemistry method at each time point. Data was analyzed using SPSS version 22. Non-parametric tests were used for paired analysis and two groups’ comparison. Spearman’s rank test was used for correlation analysis. A Chi-square test was used for categorical variables. A *p*-value < 0.05 was considered statistically significant.

**Results:**

Before the start of treatment, plasma levels of ferritin were raised in 13% and 45%, CRP in 21% and 64%, and ADA in 70% and 60% of TB lymphadenitis and pleuritis cases respectively. Levels of all three biomarkers with raised values at baseline decreased significantly with treatment at both 2 and 6 months in all patients. [Ferritin (2 months *p* = 0.001, 6 months *p* < 0.001), CRP (2 months *p* < 0.001, 6 months *p* < 0.001), ADA (2 months *p* = 0.039, 6 months *p* < 0.004)]. Plasma levels of ferritin (median 300 ng/ml range = 145–758 ng/ml) and CRP (median 11.73 mg/L, range = 10.45–17.84 mg/L) were significantly higher in TB pleuritis patients, while the levels of ADA were not significantly different among the two groups. Biosignatures generated by different combinations showed that a combination of all three biomarkers could predict treatment response in 83% and 100% of all patients at 2 and 6 months of treatment respectively.

**Conclusion:**

A combination of serum ferritin, CRP, and ADA shows a promising role in monitoring response to treatment in TB lymphadenitis and TB pleuritis patients. Similar studies in larger cohorts are needed to establish a definite role of these biomarkers in EPTB patients.

## Background

Tuberculosis (TB) remains one of the leading infectious causes of death in the world, however, if diagnosed and treated effectively, the disease is mainly curable [1, 2]. According to the World Health Organization (WHO) global report 2023, there were about 10.6 million TB patients worldwide in 2022 [3]. TB typically affects the lungs but can also affect any other site in the body, leading to the development of extrapulmonary TB (EPTB). Pleura and lymph nodes are the two most common sites affected in the majority of EPTB patients [4, 5]. EPTB is estimated to contribute to 24% of all notified TB cases in the world [6]. According to a nationwide survey conducted in Pakistan, EPTB cases accounted for 29% of all notified TB cases in 2019 [7]. EPTB poses a significant diagnostic challenge and treatment is often started without bacteriological confirmation [8]. Therefore, monitoring response during treatment is extremely important as these patients undergo a prolonged administration of multiple drugs and confirmation of diagnosis, decision to continue, and the exact duration of treatment often depends on the patient’s response to treatment [2]. Even in bacteriologically confirmed EPTB cases repeated sampling during treatment is not always possible, and response to treatment often relies on clinical criteria [9, 10]. Currently, there is a lack of reliable objective criteria that can be used routine clinical practice for monitoring response to treatment in EPTB to indicate cure or failure [11–13]. We previously proposed a five-biomarker biosignature from unstimulated plasma of EPTB patients for monitoring response to treatment using a multiplex platform [2], but this technique cannot be performed in routine laboratories. This study aimed to evaluate the role of common inflammatory biomarkers used in routine clinical laboratories such as, ferritin, CRP, and ADA to monitor response to treatment in TB lymphadenitis and TB pleuritis, the two common manifestations of EPTB.

## Methods

This prospective longitudinal cohort study was conducted at Gulab Devi Hospital, Lahore, Pakistan. The study was approved by the Institutional Review Board, Al-Aleem Medical College & Gulab Devi Educational Complex Lahore (GDEC/18–322) and Regional Committee for Medical and Health Research Ethics, Western-Norway (2018/2392/REK vest). Patients of all ages with presumptive EPTB attending the chest medicine outpatient department were enrolled from April 2016 to August 2017. This study was nested in a large research project aimed at improving the diagnosis of EPTB by the implementation of a sensitive and specific assay in routine TB diagnosis. Previously undiagnosed new EPTB cases were included in the study. Already diagnosed EPTB cases and patients refusing to participate were excluded from the study. All patients were initiated on a standard 6-month anti-TB treatment including isoniazid, rifampin, pyrazinamide, and ethambutol for the first 2 months (Intensive phase), followed by isoniazid and rifampin for 4 months (continuation phase). Patients were evaluated clinically by a physician at the end of 2 months of intensive treatment and again on completion of 6 months of treatment. Treatment was completed at 6 months in patients showing complete regression of local signs and symptoms (responders), while it was extended beyond 6 months in partial responders. Patients with extended treatment were called every 2 months till complete regression of clinical signs and symptoms and treatment was then declared completed by the physician [14, 15]. Written informed consent was obtained from all the participants before the start of the study.

### Sample collection

Blood samples (5 ml) were collected at 0 months (baseline), 2 months, and 6 months after treatment using EDTA vacuum blood collection tubes (Vacutainer). Plasma was separated by centrifugation for 10 min at 1000 g, and plasma samples were stored initially at − 20^0^C and then shifted to − 80^0^C until use [16]. For patients having enlarged lymph nodes, an excision biopsy was performed and the sample was sent for histopathology and microbiological examination. For patients with pleural effusions, aspirated fluids were sent for cytology and microbiological workup.

### Processing of samples

The lymph node biopsies and aspirated pleural fluids were processed by the universal sample processing method and the sediment was used for smear examination for acid-fast bacilli by Ziehl–Neelsen and auramine stain, Xpert MTB/RIF assay (Xpert), and mycobacterial cultures on solid (Lowenstein Jensen) and liquid media (Mycobacteria Growth Indicator Tube 960TM; Becton Dickinson, Sparks, MD, USA) [17]. Auramine O-stained smears were examined using a light-emitting diode fluorescence microscope [18]. Xpert was performed according to the manufacturer’s protocol [19]. Two slopes of the Lowenstein Jensen medium and one Mycobacteria Growth Indicator Tube 960TM; (Becton Dickinson, Sparks, MD, USA) were inoculated for cultures [17].

### Measurements of biomarkers (Ferritin, CRP, and ADA) in plasma

The plasma levels of ferritin and CRP were measured by an enzyme-linked immunosorbent assay (ELISA) according to the manufacturer's instructions [20, 21]. Briefly frozen plasma samples were thawed, vortexed, and centrifuged for 10 min at 10,000 g before the assays were performed [16]. Blanks and standards were run in all experiments. The test samples was allowed to react simultaneously with specific antibodies resulting in the CRP/ferritin molecule sandwiched between the solid phase mouse monoclonal anti-CRP/anti-ferritin antibody on the micro-titer wells and the enzyme-linked antibodies (goat anti-CRP/anti-ferritin antibody-horseradish peroxidase) after 45–60 min of incubation at room temperature for CRP and ferritin respectively. After washing the wells tetramethyl benzidine (TMP) was added and incubated for 20 min resulting in the development of a blue color. This was followed by the addition of a stop solution (2N hydrochloric acid solution). The absorbance was measured at 450 nm with a Human microtiter plate reader (Huma-Reader HS). The cut-off values for different biomarkers were taken as per manufacturer protocol, ferritin > 140 ng/ml for patients ≥ 15 years, 220 ng/ml for adult males, > 124 ng/ml for female patients [20], and CRP > 10 mg/L [21]. ADA levels were determined using colorimetric kinetic chemistry method as per manufacturer’s instructions (Spinreact) [22]. A 5 μl sample was mixed with 180 μl of reagent R1(purine nucleoside phosphorylase (PNP), 4-amino antipyrine (4-AA), xanthine oxidase (XOD) and Tris–HCl pH 8.0) and incubated at 37ºC for 3 min. Then 90 ul of reagent R2 (Tris–HCl pH 4.0, Adenosine & N-Ethyl-N-(2-hydroxy-3- sulphopropyl) -3-methyl aniline (EHSPT)) was added into a cuvette, mixed and waited for 5 min. Initial absorbance was noted and again after 3 min. ΔA/min was recorded. ΔA/min for calibrator was also noted and the absorbance change per minute was calculated using the following formula [22].

$$\mathrm{ADA}(\mathrm{IU}/\mathrm L)\;=\frac{\triangle\mathrm{sample}/\min}{\triangle\mathrm{calibrator}/\min}\;\times\;\mathrm{Calibrator}\;\mathrm{value}$$

The cut-off value for ADA was taken as 15 U/L [22].

### Categorization of the patients

By combining clinical, radiological, and laboratory findings, we divided our patients into the following “confirmed” and "probable” TB cases.

- i) A “confirmed TB pleuritis” case when pleural fluid was positive for *Mycobacterium tuberculosis* (Mtb) on culture and/or Xpert.
- ii) A confirmed “TB lymphadenitis” case when a lymph node biopsy was positive for Mtb on culture and/or Mtb Xpert.
- iii) A “probable TB pleuritis” case was a clinically diagnosed TB pleuritis patient presenting with symptoms and laboratory findings suggestive of TB pleuritis (Lymphocytosis, a protein level of more than 3 g/dl, or concomitant PTB suggested by positive acid-fast bacilli smear and/or chest radiograph) and showing good clinical response to anti-TB treatment.
- iv) A “probable TB lymphadenitis” case was a clinically diagnosed TB lymphadenitis patient in whom symptoms, clinical findings, and histopathology were consistent with TB lymphadenitis and showed a good clinical response to anti-TB treatment.

Patients with a history of diabetes or random blood sugar levels > 200 mg/dl at the time of inclusion in the study were categorized as diabetics.

### Treatment Response

All confirmed and clinically diagnosed TB lymphadenitis and TB pleuritis patients were prescribed a standard 6-month anti-TB treatment. At each follow-up visit, treatment response was considered good if two of the following three conditions were fulfilled, i) regression of symptoms, ii) regression of local signs of disease (regression of lymph nodes in lymphadenitis patients and regression of pleural effusion on ultrasound among the pleuritis cases), iii) weight gain.

Patients were categorized into two groups based on their clinical response on follow-up visits.

i) Responders, showing a good clinical response, ii) Partial responders, showing some improvement but with persistent clinical signs and symptoms.

After follow-up at 2-month, the continuation phase (isoniazid and rifampin) was started in both groups. Treatment was completed at 6 months in patients showing complete regression of local signs and symptoms (responders), while it was extended beyond 6 months in partial responders. Patients with extended treatment were called every 2 months till complete regression of clinical signs and symptoms and treatment was then declared completed by the physician.

### Statistical analysis

Data was analyzed using International Business Machine (IBM) – Statistical Package for Social Sciences (SPSS) version 22. Wilcoxon signed-rank test was used to compare paired observations at different time points. The chi-square test was used for categorical data. Mann–Whitney U test was used for 2 groups’ comparison. Correlation analysis was done with Spearman’s rank test. A *p*-value < 0.05 was considered statistically significant.

## Results

### Patients’ characteristics

A total of 92 EPTB patients registered at Gulab Devi Hospital for anti-TB treatment were included in the study. Table 1 shows the demographic and clinical characteristics of the study population. There were 49 (53%) TB lymphadenitis and 43 (47%) TB pleuritis patients. There were significantly more females (73%) among lymphadenitis patients while the males (70%) were in the majority of TB pleuritis patients. There were 4/82 (5%) patients with a history of having diabetes. There were significantly more TB pleuritis patients giving history of weight loss (*p* = *0*.013), loss of appetite (*p* < 0.001), and night sweats ((*p* = *0*.037) as compared to TB lymphadenitis patients. TB pleuritis patients presented more frequently with systemic symptoms than lymphadenitis patients (*p* < 0.001). The majority of lymphadenitis (82%) cases were of confirmed TB as compared to (28%) pleuritis cases. After 2 months of treatment significantly more (*p* = 0.001) pleuritis patients 31/43 (72%) showed good response to treatment (responders) as compared to the lymphadenitis 17/47 (36%) patients. The remaining patients showed some improvement, but all symptoms did not fully resolve (partial responders). After 6 months of treatment, 44/47 lymphadenitis patients and 41/43 pleuritis patients came for the follow-up visit. Treatment was declared completed for most of the patients at 6 months, while 19 (15 lymphadenitis and 4 pleuritis) patients were declared partial responders at 6 months and needed extension of treatment till complete resolution of symptoms and clinical signs. All patients demonstrated clinical improvement at the end of treatment.

**Table 1:** Demographic and clinical characteristics of the study population

**Patient characteristics**	**TB Lymphadenitis**	**TB Pleuritis**	*P* -value*
	*N*** = 49**	*N*** = 43**	
**Age in years**			
< 15 years, n (%)	09 (18)	0 (0)	*p* = 0.003
≥ 15 years, n (%)	40 (82)	43(100)	
**Gender, n (%)**			
Male	13(27)	30 (70)	*p* < 0.001
Female	36 (73)	13 (30)	
**Symptoms, n/N (%)**			
Fever	35/44 (80)	31/36 (86)	*p* = 0.354
Weight loss	10/44 (23)	16/35 (46)	*p* = 0.013
Loss of appetite	12/44 (27)	24/35 (69)	*p* < 0.001
Night sweats	04/44 (09)	09/35 (26)	*p* = 0.037
Fatigue	23/43 (53)	25/36 (69)	*p* = 0.086
**Combinations of symptoms**			
** ≤ **2 symptoms	30/44(68)	12/37 (32)	*p* < 0.001
≥ 3 symptoms	14/44(32)	25/37 (68)	
**HIV status, n (%)**			
Positive	0 (0)	0 (0)	
Negative	49(100)	43 (100)	
**History of Diabetes, n/N(%)**			
Yes	01/45 (02)	03/37 (08)	
No	44/45 (98)	34/37 (92)	
**Patient Categorization, n/N (%)**			
Clinically diagnosed TB	09/49 (18)	31/43 (72)	
Confirmed TB	40/49 (82)	12/43 (28)	*P* < 0.001
Culture positive	12/40 (30)	09/12 (75)	
Xpert positive	03/40 (08)	01/12 (08)	
Culture + Xpert positive	25/40 (63)	02/12 (17)	
**Treat response at 2 M n/N(%)**			
Responders	17/47 (36)	31/43 (72)	*P* = 0.001
Partial responders	30/47 (64)	12/43 (28)	
**Treatment response at 6 M/end of the treatment**^a^** n/N(%)**			
Responders	44/44 (100)	41/41 (100)	
Partial responders	0/44(0)	0/44 (0)	

*N* Total number, *n* number, %: percentage, *TB* tuberculosis, *M* month, *HIV* Human immunodeficiency virus

^*^Calculated using chi-square tests

^a^Treatment was extended for 19 (15 lymphadenitis and 4 pleuritis) patients

### Biomarkers at baseline (before treatment)

**Fig. 1:**
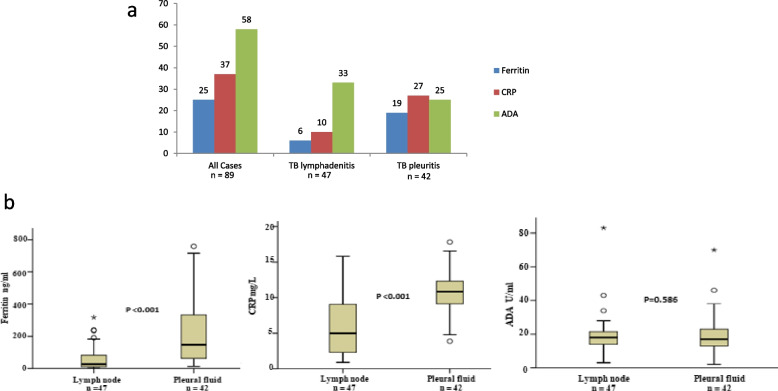
Plasma levels of ferritin, CRP, and ADA in all EPTB, TB lymphadenitis and TB pleuritis patients at baseline before treatment. a) Bar graphs showing number of patients with raised levels of biomarkers at baseline. b) Box plots showing plasma levels of biomarkers in TB lymphadenitis and TB pleuritis patients at baseline. Mann–Whitney U test was used to analyze the difference in levels of biomarkers in both groups. A p value < 0.05 was considered significant. Boxes represent the median and interquartile range, and the whisker shows minimum/maximum values. TB: Tuberculosis, ADA: Adenosine deaminase, CRP: C-reactive protein, n = number of patients

Figure 1a shows number of the patients with raised levels of biomarkers at the start of the treatment. Plasma samples were available for 89 (47 TB lymphadenitis and 42 TB pleuritis) patients at the start of the treatment. The levels of ferritin were raised in 25/89 (28%), CRP in 37/89 (42%), and ADA in 58/89 (65%) of all EPTB cases as compared to the normal reference values. In patients with TB lymphadenitis, ferritin levels were raised in 6/47(13%), CRP in 10/47 (21%), and ADA in 33/47 (70%) of cases. In patients with TB pleuritis, ferritin levels were found to be raised in 19/42 (45%), CRP in 27/42 (64%), and ADA in 25/42 (60%) of the cases. Taking all three biomarkers together, levels of anyone of these markers were raised in 79% and 90% of TB lymphadenitis and TB pleuritis patients respectively.

Figure 1b shows difference in plasma levels of biomarkers among TB lymphadenitis and TB pleuritis patients, levels of ferritin (*p* < 0.001) (median 300 ng/ml range = 145–758 ng/ml) and CRP (*p* < 0.001) (median 11.73 mg/L, range = 10.45–17.84 mg/L) were significantly higher in TB pleuritis patients, while the levels of ADA with a median of 21U/L (range = 16–83 U/L) were not significantly different among the two groups.

Next the levels of biomarkers were compared among the culture-positive and negative cases with the assumption that the culture-positive cases would have a higher bacterial load.

**Fig. 2:**
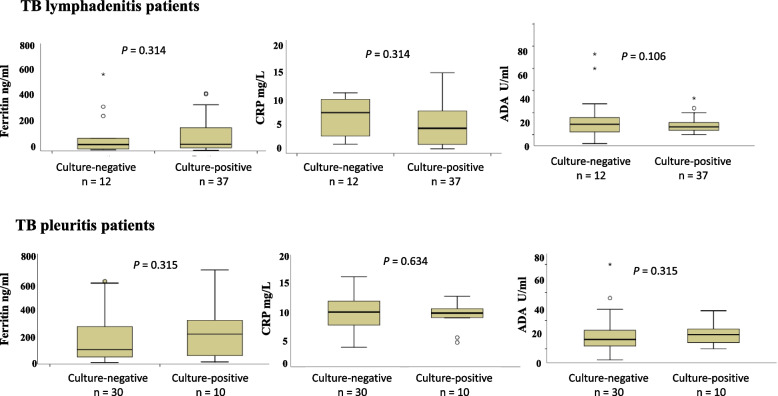
Box plots showing comparison of plasma levels of ferritin, CRP, and ADA between culture-positive and culture-negative TB lymphadenitis and TB pleuritis patients. The Mann Whitney U test was used to compare biomarkers expression in both groups. A *p* < 0.05 was considered significant. Boxes represent the median and interquartile range, and the whisker shows minimum/maximum values. TB: Tuberculosis, ADA: Adenosine deaminase, CRP: C-reactive protein, n = number of the patients

Figure 2 shows difference in plasma levels of biomarkers between culture-positive and culture-negative cases. Among TB lymphadenitis patients plasma samples were available for 12 culture-negative and 37 culture-positive patients. The levels of ferritin (*p* = 0.314), CRP (*p* = 0.314), and ADA (*p* = 0.106) were not significantly different among culture-positive and culture-negative cases. Among TB pleuritis patients plasma samples were available for 30 culture-negative and 10 culture-positive patients. The levels of ferritin (*p* = 0.315), CRP (*p* = 0.634), and ADA (*p* = 0.315) were not significantly different among culture-positive and culture-negative cases.

### Association of biomarkers with symptoms burden at baseline

**Fig. 3:**
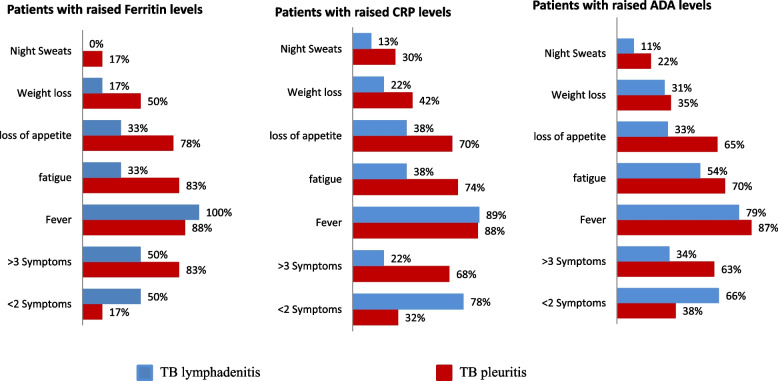
Bar graphs showing distribution of different constitutional symptoms among TB lymphadenitis and TB pleuritis patients with raised plasma levels of ferritin, CRP, and ADA. TB: Tuberculosis, ADA: Adenosine deaminase, CRP: C-reactive protein

Figure 3 shows the number of TB lymphadenitis and TB pleuritis patients with raised levels of biomarkers and their association with different constitutional symptoms.

Among TB lymphadenitis patients with raised ferritin, CRP, and ADA levels, a history of having ≤ 2 constitutional symptoms were reported by 50%, 78%, and 66%, while a history of having ≥ 3 constitutional symptoms were reported by 50%, 22%, and 34% patients respectively. When constitutional symptoms were analyzed separately, history of having fever was given by 100%, 89%, and 79% of patients; fatigue was documented in 33%, 38%, and 54%, loss of appetite in 33%, 38%, 33%, loss of weight in 17%, 22%, and 31%, and night sweats in 0%, 13%, and 11% of patients with raised levels of ferritin, CRP, and ADA respectively.

Among TB pleuritis patients with raised ferritin, CRP, and ADA levels, a history of having ≤ 2 constitutional symptoms was reported by 17%, 32%, and 38%, while a history of having ≥ 3 constitutional symptoms was given by 83%, 68%, and 63% patients respectively. When constitutional symptoms were analyzed separately, history of having fever was given by 88%, 88%, and 87% of patients, fatigue was documented in 83%, 74%, and 70%, loss of appetite in 78%. 70%, and 65%, loss of weight in 50%, 42%, and 35%, and night sweats in 17%, 30%, and 22% of patients with raised levels of ferritin, CRP, and ADA respectively.

A clear tendency towards an increase in levels of all 3 biomarkers with high symptom scores was seen among TB pleuritis patients as compared to the TB lymphadenitis patients. Among all symptoms, fever was most associated with raised biomarker levels.

**Fig. 4:**
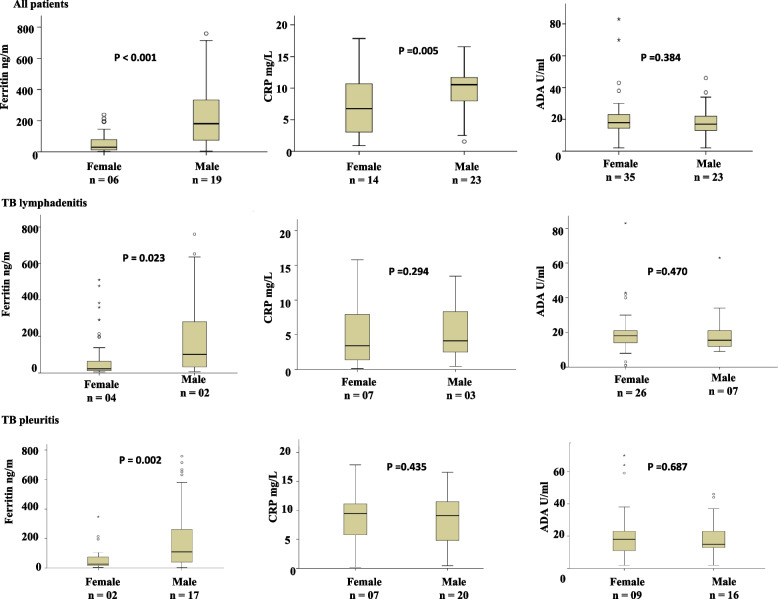
Box plots showing comparison of raised plasma levels of ferritin, CRP, and ADA between female and male extrapulmonary tuberculosis patients at baseline before start of the treatment. The Mann Whitney U test was used to compare biomarkers expression in both groups. A *p* < 0.05 was considered significant. Boxes represent the median and interquartile range, and the whisker shows minimum/maximum values. TB: Tuberculosis, ADA: Adenosine deaminase, CRP: C-reactive protein, n = number of the patients

Figure 4 shows the difference in levels of biomarkers among male and female patients. In all EPTB patients, plasma levels of ferritin (*p* < 0.001) and CRP (*p* = 0.005) were found to be significantly raised in male patients whereas, no significant difference was observed in plasma levels of ADA (*p* = 0.384) between male and female patients. When analyzed separately, in TB lymphadenitis patients, ferritin levels were found to be significantly raised (p = 0.023) in male patients as compared to the females, Whereas, no significant difference was observed in plasma levels of CRP (*p* = 0.294) and ADA (*p* = 0.470) among male and female patients. Similar findings were seen among TB pleuritis patients in whom ferritin levels were found to be significantly raised (*p* = 0.002) in male patients as compared to the females whereas, no significant difference was observed in plasma levels of CRP (*p* = 0.435) and ADA (*p* = 0.687) between male and female patients.

### Changes in levels of biomarkers with treatment

**Fig. 5:**
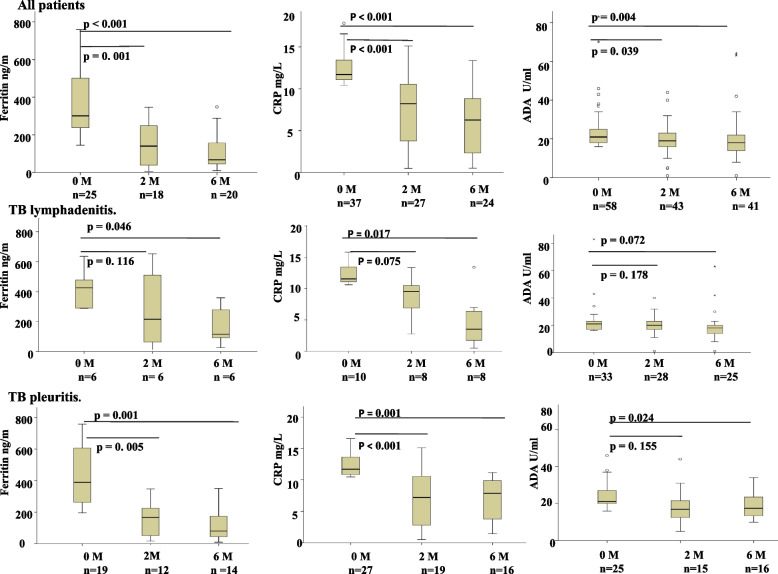
Box plots showing changes in plasma levels of ferritin, CRP, and ADA at 2 and 6 months of treatment for extrapulmonary tuberculosis patients having raised values at baseline. The Wilcoxon signed rank test was used to compare biomarkers expression at different time points. A *p* < 0.05 was considered significant. Boxes represent the median and interquartile range, and the whisker shows minimum/maximum values. n = number of patients at different time points. M: month of treatment, ADA: Adenosine deaminase, CRP: C-reactive protein,

Figure 5 shows plasma levels of biomarkers at 2 and 6 months of treatment as compared to the baseline values. Levels of all three biomarkers with raised values at baseline decreased significantly with treatment at both 2 and 6 months. [Ferritin (2 months *p* = 0.001, 6 months *p* < 0.001), CRP (2 months *p* < 0.001, 6 months *p* < 0.001), ADA (2 months *p* = 0.039, 6 months *p* < 0.004)]. When analyzed separately, levels of ferritin (*p* = 0.046) and CRP (*p* = 0.017) decreased significantly only at 6 months of treatment, while, ADA levels did not decease at any time point among TB lymphadenitis patients. In TB pleuritis patients levels of ferritin (2 months *p* = 0.005, 6 months *p* = 0.001), and CRP (2 months *p* < 0.001, 6 months *p* = 0.001) decreased at both time points, however, ADA levels decreased only at 6 months (*p = *0.024) of treatment.

### Correlation between biomarkers

Table 2 shows the correlations among biomarkers at baseline and during treatment. Among all patients, ferritin and CRP levels showed a significant positive correlation at baseline (r = 0.340, *p* = 0.001), at 2 months (r = 0.406, *p* = 0.001), and 6 months (r = 0.234, *p* = 0.048) of treatment. ADA levels did not show any significant correlation with ferritin or CRP at baseline or any other time point during treatment in these patients. When analyzed separately, TB lymphadenitis patients showed a significant positive correlationonly between CRP and ADA (r = 0.375, *p* = 0.009) levels at baseline, whereas: no significant correlation was seen between these biomarkers at 2 and 6 months. Among TB pleuritis patients, no significant correlation was observed between ferritin, CRP, and ADA levels at baseline, however, a significant positive correlation was seen between ferritin and CRP (r = 0.454, *p* = 0.020) at 2 months. No significant correlation was seen between ferritin, CRP, and ADA levels at 6 months among TB pleuritis patients.

**Table 2:** Correlation coefficients showing associations between the serum ferritin, CRP, and ADA levels among TB lymphadenitis and TB pleuritis patients

**All patients**						
	Ferritin 0 M	ADA 0 M	Ferritin 2 M	ADA 2 M	Ferritin 6 M	ADA 6 M
CRP 0 M	0.338^b^	0.143				
ADA 0 M	-0.078					
CRP 2 M			0.406^b^	0.120		
ADA 2 M			-0.052			
CRP 6 M					0.234^a^	0.085
ADA 6 M					-0.167	
**TB lymphadenitis**						
	Ferritin 0 M	ADA 0 M	Ferritin 2 M	ADA 2 M	Ferritin 6 M	ADA 6 M
CRP 0 M	0.166	0.375^b^				
ADA 0 M	-0.105					
CRP 2 M			0.093	0.075		
ADA 2 M			-0.108			
CRP 6 M					0.132	-0.057
ADA 6 M					-0.176	
**TB pleuritis**						
	Ferritin 0 M	ADA 0 M	Ferritin 2 M	ADA 2 M	Ferritin 6 M	ADA 6 M
CRP 0 M	-0.011	0.007				
ADA 0 M	-0.014					
CRP 2 M			0.454^b^	0.288		
ADA 2 M			0.138			
CRP 6 M					0.231	0.230
ADA 6 M					-0.183	

The Spearman correlation test was used to see the association among biomarkers

*ADA* Adenosine deaminase, *CRP* C-reactive protein, *M* Month of treatment, *TB* Tuberculosis

^a^Correlation is significant at the 0.05 level (2-tailed)

^b^Correlation is significant at the 0.01 level (2-tailed)

### Changes in levels of biomarkers with treatment among responders and partial responders at 2 months of treatment

**Fig. 6:**
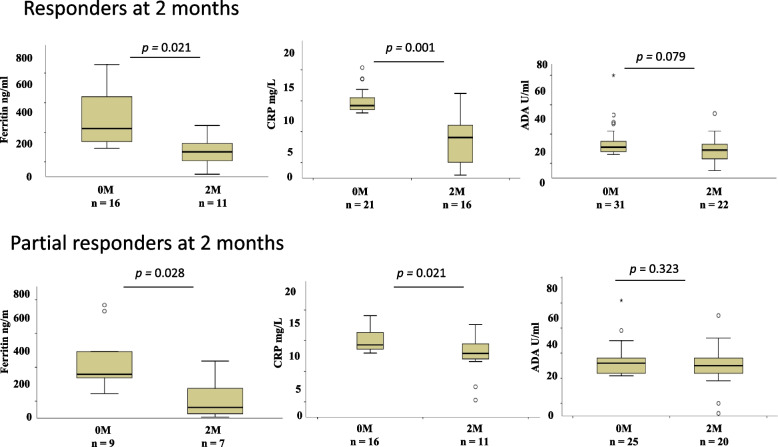
Box plots showing changes in plasma levels of ferritin, CRP, and ADA at 2 months of treatment in responders, and partial responders having raised values at baseline. The Wilcoxon signedrank test was used to compare biomarkers expression at two time points. A *p* < 0.05 was considered significant. Boxes represent the median and interquartile range, and the whisker shows minimum/maximum values. N = number of patients at different time points. M: month of treatment, ADA: Adenosine deaminase, CRP: C-reactive protein, n = number of patients

Figure 6 shows changes in the levels of biomarkers among responders and partial responders at 2 months of treatment in patients with raised levels of biomarkers at baseline. Among responders, both ferritin (*p* = 0.021) and CRP (*p* = 0.001) decreased significantly while, no significant change was seen in ADA levels (*p* = 0.079) at 2 months of treatment. Among partial responders levels of ferritin (*p* = 0.028) and CRP (*p* = 0.021) decreased significantly with treatment at 2 months of treatment as compared to the baseline levels, while, no change was observed in plasma levels of ADA (*p* = 0.323) with treatment.

### Biosignatures predicting response to treatment

Table 3 shows various combinations of biomarkers with their sensitivities in predicting treatment response at different time points. Biosignatures were synthesized based on combinations of biomarkers changing in a maximum number of patients with treatment. A decrease in levels of any biomarker included in the biosignature was associated with a favorable treatment response. Changes in any of three biomarkers (Ferritin, CRP, and ADA) could predict treatment response in 94% of patients at 2 and 100% of patients at 6 months of treatment among all cases.

**Table 3:** Combinations of biomarkers with their sensitivities in predicting treatment response at second and six months of treatment, and the sensitivity of the biosignatures in predicting treatment response

Biomarkers	All Patients		TB lymphadenitis		TB Pleuritis	
	**0 M-2 M n/N (%)**	**0 M-6 M n/N (%)**	**0 M-2 M n/N (%)**	**0 M-6 M n/N (%)**	**0 M—2 M n/N (%)**	**0 M—6 M n/N (%)**
Ferritin	13/18 (72)	15/18 (83)	04/6 (67)	05/6 (83)	10/11 (91)	13/13 100)
CRP	18/27 (67)	19/24 (79)	05/8 (63)	07/8 (88)	13/19 (68)	13/17 (76)
ADA	9/43 (21)	16/41 (39)	04/28 (14)	10/25 (40)	05/15 (33)	06/16 (38)
Ferritin + CRP	15/18 (83)	18/18 (100)	05/06 (83)	05/05 (100)	11/11 (100)	13/13 (100)
Ferritin + ADA	17/18 (94)	16/17 (94)	06/06 (100)	05/06 (83)	10/10 (100)	12/12 (100)
CRP + ADA	22/29 (76)	20/24 (83)	07/08 (88)	07/08 (88)	14/17 (82)	14/16 (88)
Ferritin + CRP + ADA	17/18 (94)	18/18 (100)	06/06 (100)	06/06 (100)	11/11 (100)	13/13 (100)

*M* month of treatment, *TB* tuberculosis, *N* Total number with raised values, *n* number of patients showing decrease with treatment, *ADA* Adenosine deaminase, *CRP* C-reactive protein

## Discussion

We assessed the levels of ferritin, CRP, and ADA in unstimulated plasma of TB lymphadenitis and pleuritis patients at baseline and changes in their levels with anti-TB treatment to see their utility as biomarkers to monitor treatment response. The levels of ferritin, CRP, and ADA were found to be raised as compared to the standard reference values in only a proportion [25/89 (28%), 37/89 (42%), and 58/89 (65%), respectively] of our cohort of EPTB patients at baseline. The finding of raised levels of these biomarkers in EPTB patients signifies their role as potential biomarkers for predicting treatment response in these patients. When the levels were tested during anti-TB treatment and compared with baseline levels, a significant decrease was seen in the levels of all three biomarkers at 2 [Ferritin (*p* = 0.001), CRP (*p* < 0.001), and ADA (*p* = 0.039), and 6 months (*p* < 0.001), CRP (*p* < 0.001), ADA *p* < 0.004) of anti-TB treatment. A combination of all three biomarkers was found to be raised in a higher proportion (84%) and changes in their levels could predict response to treatment in 94% of patients after 2 months and 100% of patients after 6 months of treatment among all EPTB cases.

Recent studies have evaluated the role of multiple biomarkers in TB using the multiplex technique providing encouraging results, however, these biomarkers are not available for routine clinical use [2, 11, 23, 24]. Furthermore, most of the studies demonstrating raised levels of ferritin, CRP, and ADA in TB and a decrease with treatment have focused on pulmonary TB [11, 24–28] with relatively few reports on these markers in EPTB cases [11, 27, 29]. Ferritin levels are often found to be increased in infections as a result of alterations in the host’s iron metabolism induced by the invading pathogens [30]. We found increased levels of ferritin in 13% of TB lymphadenitis and 45% of TB pleuritis cases, indicating that levels are different in different manifestations of EPTB. A study from India evaluating acute phase proteins in the serum of pulmonary TB and TB lymphadenitis patients found elevated serum CRP levels (median: 1692 pg/ml or 16.92 mg/L) in 44 TB lymphadenitis patients as compared to the healthy controls. Almost all patients included in the study had levels more than 10 mg/L [31], while we found raised levels of CRP (> 10 mg/L) in only 21% of 49 TB lymphadenitis patients with a lower median of 11.55 mg/L. Most of the studies on acute phase proteins usually give mean or median levels with little information on exact numbers or percentages of patients with raised plasma levels of these biomarkers. Chendi et al. proposed a 5-biomarker signature including CRP and ferritin for the diagnosis of pulmonary and EPTB from two settings, a TB non-endemic area (Norway) and a TB endemic area (South Africa). Interestingly, the sensitivity of biomarkers was different in different epidemiological settings, working better in TB non-endemic area. The sensitivity was also found to be low in the 39 EPTB cases included in the study, however the exact sites of EPTB cases were not specified by the authors [11]. It was shown that ADA levels from cerebrospinal fluids of patients diagnosed with TB meningitis were higher than those diagnosed with bacterial meningitis [32]. We could not find many studies with levels of these biomarkers in different types of EPTB, this underlines the need for more studies with the inclusion of different types of TB to clearly understand the role of acute phase proteins in EPTB.

In our study, the levels of ADA were raised in a higher proportion of patients than CRP and ferritin. Previous studies have also reported raised serum ADA levels in pulmonary TB and EPTB, however, information about the proportion of cases with raised levels is not always given [33–36]. Two studies from India, in agreement with our study, showed raised ADA levels among 83% and 72% of TB lymphadenitis patients [37, 38]. In contrast, another study from Bangladesh demonstrated raised serum ADA levels in only 4/10 (40%) TB lymphadenitis and 34/36 (94%) of TB pleuritis patients [39], however, the low number of lymphadenitis patients could explain the smaller proportion with raised ADA levels in this study.

Raised plasma ferritin and CRP levels returned to normal levels with treatment in the majority (83% and 79% respectively) of our patients while the levels of ADA normalized only among 39% of cases. In a study on 26 patients with spinal TB, only 15 remained in follow-up at 12 months and reported a significant decrease in serum CRP and ferritin (*p* < 0.01) throughout treatment [40]. Another study on 44 TB lymphadenitis patients has also shown that CRP levels decreased significantly from a median of 1692 pg/ml (16.92 mg/L) to 1398 pg/ml (13.98 mg/L) with treatment [31]. Raised ADA levels have been also reported to decrease with successful treatment in pulmonary TB patients [41–43], however, there is a paucity of literature on the role of ADA in monitoring treatment response in EPTB. We could find only one study including 51 EPTB patients showing a significant decrease in serum ADA levels with treatment [44]. However, the decrease in ADA levels in different types of EPTB is not mentioned. In our study when analyzed separately no significant change was observed in levels of ADA with treatment in TB lymphadenitis patients whereas among TB pleuritis patients ADA levels significantly decreased only at 6 months of treatment indicating that biomarkers may behave differently in different manifestations of TB. The patients’ response to infection and treatment would depend on a variety of host and bacteriological factors and is expected to vary among individuals and between different disease sites. It is not exactly known why Mtb causes indolent lymphadenitis in one patients and life threatening meningitis in others. Study of different host biomarkers can help us understand the different immune pathologies induced by Mtb at different body sites. To our knowledge, ours is the first study to address ADA as a prognostic marker in different types of EPTB.

We did not find any correlation between bacterial load and raised plasma biomarkers, which is in agreement with a study on pulmonary TB where no significant difference was found in CRP and ferritin levels among patients with culture-positive and negative pulmonary TB cases [25, 45]. However, a study from Pakistan on children demonstrated significantly increased ferritin and CRP levels in confirmed pulmonary TB as compared to clinically diagnosed (probable and possible) pulmonary TB cases [46]. This disparity could be due to the over-diagnosis of TB in children as there is variable clinical presentation and childhood TB is usually difficult to diagnose. On the other hand, these findings suggest that the inflammation in response to Mtb is independent of the microbial burden. EPTB is a paucibacillary disease and the disease manifestations and severity is usually dependent on the exaggerated host immune response to a relatively low bacterial load. More studies with different types of EPTB in adults and pediatric populations are needed to study the association between bacterial load and inflammatory markers.

When levels of biomarkers were compared between TB lymphadenitis and TB pleuritis cases, it was seen that ferritin (*p* < 0.001) and CRP (*p* < 0.001) levels were significantly higher in TB pleuritis cases as compared to TB lymphadenitis cases, whereas ADA levels were not significantly different among the two groups. TB pleuritis patients had more constitutional symptoms than TB lymphadenitis correlating with higher disease severity and probably a higher degree of systemic inflammation explaining higher CRP and ferritin levels. It has been demonstrated before that CRP levels were raised in pulmonary TB patients with cavitary, bilateral disease, and a positive acid-fast bacilli sputum smear as compared to non-cavitary, less extensive disease and patients with no acid-fast bacilli detected in sputum smears indicating that CRP levels can be used as a marker of disease severity of TB [47]. Similarly, ferritin has been seen to be associated with disease severity and sputum positivity in pulmonary TB patients [48]. Raised ferritin and CRP levels in PTB have also been seen to be associated with an increased risk of pulmonary TB-related ischemic stroke [49]. Another study also reported a significant correlation between ferritin and CRP with disease severity in pulmonary TB [48]. However, there is a lack of studies on EPTB to show an association of these two biomarkers with disease severity. Our study adds to the evidence that raised ferritin and CRP levels are indicators of disease severity in EPTB patients. ADA, on the other hand, was not different between TB lymphadenitis and pleuritis cases, in confirmation with another study evaluating ADA levels in different types of TB found no significant difference among ADA levels in pulmonary TB and EPTB [50], or among different types of EPTB [51, 52]. In contrast, a recent study from Bengal reported significantly raised ADA levels in TB pleuritis as compared to TB lymphadenitis [39]. This difference might be because of a smaller number of ten TB lymphadenitis cases as compared to 37 TB pleuritis cases.

We demonstrated that levels of CRP and ferritin correlated positively with each other at baseline, 2, and 6 months of treatment indicating that both are good biomarkers to study the degree of inflammation. Evidence of a positive association between CRP and ferritin levels in various inflammatory diseases is previously documented [53, 54].This correlation has also been observed in pulmonary TB patients [25]. When analyzed separately CRP and ADA correlated positively with each other at baseline in TB lymphadenitis patients, but no significant correlation was seen between these biomarkers at 2 and 6 months. This was expected as ADA levels did not decrease with treatment in lymphadenitis patients. There are limited studies on correlation of biomarkers in EPTB and these findings underline the need to conduct more studies with inclusion of different types of EPTB.

Sex hormones including estrogen and testosterone have direct effects on immune cell function [55–57]. These biological gender differences in immunity may indicate that men are more vulnerable to infection while women are potentially “protected” by a robust immune response [55]. This may explain the male predominance in a relatively more severe TB pleuritis as compared to the indolent form of TB lymphadenitis and raised plasma levels of CRP and ferritin. In this study, the raised levels of ferritin were significantly higher in male patients as compared to CRP and ADA, while one study published significant race and gender differences in the population distribution of CRP among cardiovascular patients and reported female predominance [58]. We did not find any association between raised ADA levels and the gender of the patients. Our findings are consistent with a previous study including 50 pulmonary TB and 25 EPTB patients with no significant difference in ADA levels among male and female TB patients [59].

The host response to infection is multifaceted with broad variation between individuals; therefore, a single biomarker is not expected to change in all patients, whereas a combination of biomarkers could predict treatment response with reasonable certainty in more patients. It has been reported that a biosignature including two more biomarkers increases the sensitivity of predicting treatment response [2, 11, 60]. A change in any one of the biomarkers (CRP, ferritin, and ADA) included in our study predicted treatment response in 83% of our study patients at 2 months and 100% at 6 months after treatment. These biomarkers are relatively low cost and performed routinely in most clinical laboratories, making it feasible to include them in routine care to monitor response to treatment in TB lymphadenitis and TB pleuritis. If these biomarkers are integrated into the routine clinical practice and TB control programs at national levels in low-resource high TB-endemic countries, they can help in clinical decisions regarding patient care.

Our study has some limitations, i) no control group was included as we did not intend to evaluate the diagnostic accuracy of these biomarkers, ii) the absence of bacteriological confirmation in all patients due to the paucibacillary nature of EPTB. This may lead to over diagnosis of TB in some patients, however all clinically diagnosed cases included in our study showed improvement with anti-TB treatment and our findings still hold significant value and can be used to monitor response to treatment in EPTB patients. These biomarkers are point-of-care, cheap, and easily interpretable, which makes them particularly useful, in TB-endemic areas.

## Conclusions

Our findings support a multi-biomarker approach to monitor treatment response in TB lymphadenitis and TB pleuritis patients. Plasma ferritin and CRP decreased with successful treatment; however, they were raised in only a small proportion of cases. Plasma ADA levels though raised in most cases, decreased only in a small proportion of patients. These findings suggest a combination of serum ferritin, CRP, and ADA shows a promising role in monitoring response to treatment in EPTB patients. Measuring levels of these biomarkers at baseline and during treatment can help better management of these patients. There is a need for further studies to validate these results in larger population of patients including different types of EPTB patients to improve the reliability and robustness of the results. This can help find better biomarkers that can monitor response to treatment in all types of TB helping in making better clinical decisions.

## Data Availability

The datasets used during the current study is available from the corresponding author on reasonable request.

## Abbreviations

TB: Tuberculosis
WHO: World Health Organization
EPTB: Extrapulmonary TB
CRP: C-reactive protein
ADA: Adenosine deaminase
Xpert: Xpert MTB/RIF assay
